# Attempts to purchase misoprostol online in Indonesia: a mystery client study design

**DOI:** 10.1186/s12905-022-01889-6

**Published:** 2022-08-05

**Authors:** Ann M. Moore, Jesse Philbin, Iwan Ariawan, Meiwita Budiharsana, Geby Hasanah Jorgy, Zahra Izza Arifa, Akinrinola Bankole

**Affiliations:** 1 Research Division, Guttmacher Institute, 125 Maiden Lane, 7th Floor, New York, NY 10038 USA; 2 Reconstra Utama Integra, Jakarta, Indonesia; 3 Department of Biostatistics and Population, Faculty of Public Health, University of Indonesia, Depok, Indonesia

**Keywords:** Descriptive study, Medical demography, Pharmacology, Health services research, Maternal health

## Abstract

**Background:**

Abortion is highly restricted in Indonesia; self-administered misoprostol can safely induce an abortion. Brick and mortar pharmacies, a common place to purchase misoprostol off-label in other parts of the world, are monitored closely by the government authority in Indonesia which controls drugs so that they cannot function outside the law without risking arrest and prosecution. An online marketplace has sprung up in response that sells misoprostol through in-country distributors. Such procurement offers a level of safety and anonymity to the buyer and seller. So as to understand online access to misoprostol, we created a protocol to identify the most visible universe of sellers.

**Methods:**

We carried out a mystery client methodology to replicate the experiences of women procuring misoprostol online. Our study consisted of five stages: (1) identify the universe of online sellers using the most common search terms, drawn from multiple platforms to capture diversity in interactions as well as products sold (2) remove duplicates across sites as determined by their telephone numbers (3) draw a roughly probability proportional to size sample (4) contact sellers as mystery clients through text/chat, depending on the platform, and engage with them and (5) attempt to purchase drugs offered by the seller. Descriptive statistics are presented.

**Results:**

The listing generated 727 sites: 441 websites, 153 marketplace sellers, and 133 Instagram profiles. After removing duplicate listings, we identified 281 unique sellers. We selected all sellers with greater than 12 listings, 60% of sellers with 4–12 listings, 50% of sellers with 2–3 listings, and 40% of sellers with only one listing. Mystery clients were able to send initial messages to 110 sellers, of which 16 never responded. The interaction progressed to purchasing misoprostol with 76 sellers, 64 of whom sent drugs.

**Conclusions:**

As women seek to terminate unwanted pregnancies in legally restrictive settings, online sales of misoprostol must be considered. With the Covid pandemic constraining movement, the importance of this way of procuring misoprostol will likely become more appealing. Understanding this unregulated landscape is important if we are to try to improve women’s ability to safely conduct an abortion in highly restrictive settings.

## Background

In the past two decades, misoprostol, a drug that can successfully induce abortion at least 75% of the time [1], has become more available worldwide. In countries with restrictive abortion laws, self-use of misoprostol is a discreet method of abortion that poses far fewer risks than other methods [2]. Abortion in Indonesia is legally allowed only in medical emergencies, cases of severe foetal malformation; and cases of rape, but only up to six weeks gestation [3]. Despite the restrictive law, a 2018 study in Java, where almost 60% of Indonesia’s population lives, estimated that 1.6 million abortions took place annually [4]. Misoprostol is registered in Indonesia only for gastric ulcers. Pharmacies in the country are closely regulated so that off-label sales are risky for the pharmacist while poor governance of private hospitals means that there is ample opportunity of “leakage” of controlled medication into unregulated environments [5].

In response to this environment, in 2019, we conducted a mystery client study to investigate the online market of misoprostol in Indonesia. To be systematic in identifying and selecting sellers in an unknown environment, we looked to published literature for a protocol. However, no such protocol existed. A 2017 study in the United States presented results from purchasing misoprostol from online sellers, but employed a convenience sample of 18 sellers found through a brief web search [6]. Anticipating a much larger universe of sellers from which to choose and to employ a reproducible method for selecting our sample and engaging with the sellers, we created a protocol. This protocol is to our knowledge the first rigorous approach to identifying a sample of online sellers and purchasing misoprostol in a legally restricted context.

## Methods

This study consisted of five stages: (1) identifying the universe drawn from multiple platforms (2) removing sellers advertising on more than one site (3) draw a roughly probability proportional to size sample (4) contacting sellers as mystery clients through text/chat and (5) purchasing drugs offered by the seller. We provide below the protocol we followed for each of these steps.

Patient and Public Involvement: Patients and the public were not explicitly involved, although as abortion is not a visible experience, it’s possible that members of the study team had previously bought medication abortion drugs online.

### Identifying the universe drawn from multiple platforms

To construct our universe, it was necessary to identify as many online misoprostol sellers as we could find. We identified sellers through three platforms: (1) websites, identified through searching on Google; (2) Instagram profiles; and (3) online marketplaces (sites similar to Amazon). We included the following marketplace sites because they are the most popular e-commerce sites in Indonesia: Tokopedia (www.tokopedia.com), Bukalapak (www.bukalapak.com), Shopee (www.shopee.co.id), Lazada (www.lazada.com), and Blibli (www.blibli.com). This universe is fluid; we identified the universe at one point in time.

#### Selection of search terms

For conducting our web searches to find sellers, we chose the most popular relevant search terms on Google Trends as of August 5, 2019 (Table 1). We used the word *jual* (“sell” in Bahasa) in addition to the drug name. We also added phrases lacking specific drug names to our search term list, which were also popular according to Google Trends, and which may be employed by women who do not know the names of abortion drugs. On Instagram, we conducted searches for the terms listed in Table 1 using Instagram’s regular search function.

**Table 1:** Search terms used to list online misoprostol sellers in Indonesia, 2019

Google	Instagram	Online marketplace*
Jual misoprostol (sell misoprostol) Jual Cytotec (sell Cytotec) Jual obat aborsi (sell drug for abortion) Jual obat telat datang bulan (sell drug for period that did not come)	Jual obat aborsi (sell abortion drug) Obat aborsi (abortion drug) Obat telat (drug for being late) Obat telat datang (drug for missed period) Obat telat datang bulan (drug for period that did not come) Obat telat haid (drugs for late menstruation) Obat telat menstruasi (drug for late menstruation) Obat penggugur kandungan (drug for obstetric abortion)	All Obat aborsi Obat telat datang bulan Tokopedia, Bukalapak Misoprostol (miso-prostol, mi-so-pros-tol, miso prostol) Cytotec (cyto tec, cy-to-tec, cy-tot-ec, cy-totec, c.y.t.o.t.e.c., c-y-t-o-t-e-c) Blibli Misoprostol (no misspellings) Cytotec (cyto tec, cy-to-tec, cy-tot-ec, cy-totec, c.y.t.o.t.e.c., c-y-t-o-t-e-c) Lazada Misoprostol Cytotec Shopee Misoprostol (miso-prostol, mi-so-prostol, miso-prostol) Cytotec (cytotex, cy-to-tec, cyto-tec)

*Sellers on online marketplace sites sometimes used deliberate misspellings to avoid detection by algorithms used by the site’s user controls to find and take down illegal postings. These misspellings are listed in parentheses next to their corresponding “regular” search term, for each marketplace site we searched

Identifying sellers through marketplace sites required a slightly different strategy. Marketplace sites use algorithms to detect advertisements for illegal products; we found that many sellers add characters or deliberately misspell words to evade detection, e.g. “Cy-to-tec” or “Cyt0tec.” Each marketplace platform had slightly different sets of commonly used misspellings, which we believe is because each site uses different algorithms to flag profiles that do not comply with site regulations. The data collectors identified and searched for misspellings at their discretion, discussing their process with members of the study team and recording all search terms used.

#### Listing procedures

Six data collectors conducted the searches after receiving a one-day training. Data collectors searched for each term only once, keeping those results open for the duration of the listing. Marketplace sites were continuously refreshing so that a vendor that came up during a search sometimes no longer appeared when the fieldworker tried to list it. All searches were conducted on Google Indonesia (www.google.co.id) using the Chrome browser’s Incognito mode. Each search term yielded thousands of results. We began with the hits that appeared first, as these are the most relevant according to the Google search algorithm. The listing of websites continued until the hits were no longer relevant: either they stopped linking to misoprostol sellers or linked to the same pages as before. The Google search results turned up misoprostol advertisements buried in blog posts on sites such as www.medium.com, www.asus.com, www.weebly.com, and www.kompasiana.com; websites meant for other purposes such as www.goodreads.com; and sales sites, for example those selling colour vision sensors: www.cmucam.org.

During the listing, we excluded the following types of results: pages that were no longer active or had been taken down (i.e. “404-page not found”), sites that did not provide any means of contacting a seller, and irrelevant pages which did not advertise the sale of an abortion drug, such as news stories. The website listing also captured a big drug store chain; big drug store chains are legally allowed to sell online with a prescription. We excluded this site since it was advertising legal sales. The Google search listers also ignored any hits that led to Instagram profiles and online marketplace vendors, which we had another process for identifying.

To conduct searches in Instagram, data collectors logged in to their Instagram accounts in a Google Chrome incognito window. Searches were filtered in order of most recent posting. Most of the accounts that came up in searches were private, meaning that unless the account holder accepted to be “followed” by the buyer, the information of that account holder was not visible. The lister created a fake Instagram profile to follow these sellers; if they accepted her “follow” request by the end of the listing period, they were included in the sample. Some Instagram sellers directed prospective buyers to a website link in their profile; in these cases, we counted the website as part of the Instagram page.

Listing took place over two weeks in August 2019 and resulted in 727 hits: 441 websites, 153 marketplace sellers, and 133 Instagram profiles. For each search result, listers completed a web-based data capture form created using Open Data Kit (ODK). In addition to the information needed to identify the seller such as the URL and contact information, the data form also captured information on the site: specific brand names and types of pills advertised, asking price, the maximum gestational ages for which sellers offered abortion drugs, and any additional information about misoprostol or abortion given on each site. Once completed, each form was uploaded to a secure server hosted by SurveyCTO. In addition to filling out the listing form, data collectors also took screen captures of the landing page, and any testimonials or pictures of pills.

### Removing sellers advertising on more than one site

We identified duplicate listings using the telephone number listed, usually presented as a WhatsApp number. There was high variability in the number of separate sites a seller had. For example, eight sellers had 18 or more separate sites; the biggest seller had 51.

After removing duplicate listings, we identified 281 unique sellers. Some sellers had listings on multiple platforms, and we randomly chose one listing to “represent” each of them for the purpose of drawing a sample. Our sampling frame consisted of 147 websites, 49 Instagram accounts, and 85 marketplace sellers (Table 2).

**Table 2:** Sample of online misoprostol sellers and outcome of interactions with mystery clients, 2019

	Websites	Instagram	Marketplace	TOTAL
Universe of sellers identified in web listing	441	133	152	727
De-duplicated universe	147	49	85	281
*Sample*	*73*	*20*	*39*	*132*
Unable to reach: number inactive/website down	10	5	7	22
Sent initial message to seller	63	15	32	110
Seller did not respond to initial message	7	2	7	16
Established text exchange with seller	56	13	25	94
No purchase—seller stopped responding	1	0	1	2
No purchase—identified as duplicate prior to sale	7	2	3	12
No purchase—other reasons*	2	1	1	4
Purchased pills from seller	46	10	20	76
Purchased and did not receive pills	5	4	3	12
**Purchased and received pills**	**41**	**6**	**17**	**64**
**% of sample that resulted in purchasing and receiving pills**	**56%**	**30%**	**44%**	**48%**

*Other reasons include: too expensive, seller required a prescription, seller made mystery client feel unsafe, seller requested an in-person meeting for cash payment [7]

*Source*: From Ref [7], reproduced with permission from John Wiley & Sons

### Drawing a roughly probability proportional to size sample

Based on budgetary constraints, we aimed to have a final sample of 100 sellers with whom we were able to make contact. Without having any priori information about how likely it was going to be to establish contact with online vendors, we oversampled by 25%. We aimed to conduct probability sampling proportional to size of online presence. We wanted to be sure to include those sellers who had a larger presence and so we divided sellers into strata by size, each with an approximately even number of total listings as follows: sellers with between 4 and 12 listings; sellers with 2 or 3 listings; and sellers with only one listing. We selected all sellers with greater than 12 listings, 60% of sellers with 4–12 listings, 50% of sellers with 2–3 listings, and 40% of sellers with only one listing. We did not stratify by seller type (web, Instagram, or marketplace) since we do not know the relative market share among the three types, and some sellers were linked to listings across multiple platforms (e.g. both a website and an Instagram account).

This resulted in a sample of 128 sellers: 73 (57%) websites, 19 (15%) Instagram accounts, and 36 (28%) marketplace sellers. Sixty-six sellers (51%) had only one listing, 38 sellers (30%) had 2–3 listings, and the remaining 24 sellers (19%) had 4 or more listings, including the 8 sellers we designated as certainty units because of their exceptionally high number of listings. For comparison, in the sampling frame, 60% of sellers had a single listing, 28% had 2–3 listings, and 12% had 4 or more listings.

In case the sellers were well-connected to one another and our mystery client study was received with suspicion among a broad swath of sellers, we wanted to start contacting the sellers with the largest online presence first. We therefore organized the sample list in descending order by measure of size (number of listings linked to that seller), and instructed the mystery clients to contact the sellers in order of size. When sellers had the same bank account number and quoted the same price, or gave near-identical responses to questions, or engaged in non-simultaneous texting with fieldworkers, we treated them as duplicates and stopped engaging with them (n = 12). In response to omitting these sellers from our sample, we selected an additional four sellers to contact, which resulted in a total sample of 132 sellers: 73 websites, 20 Instagram accounts, and 39 marketplace sellers (Table 2).

### Contacting sellers as mystery clients through text/chat

The study team selected three of the fieldworkers who had conducted the listing to stay on and pose as mystery clients. We first conducted a pilot test of five sellers. The five sellers we selected were those with only one listing.

Mystery clients contacted all selected sellers using WhatsApp using SIM cards purchased exclusively for use in the study, except for 18 marketplace sellers who we were required to contact through the instant chat window hosted on the marketplace site. If no contact information was available and the seller’s URL no longer existed, that seller was marked as having invalid or missing contact information (n = 22).

Mystery clients were able to send initial messages to 110 sellers, of which 16 never responded. If the seller did not respond for 24 h after the last message, the mystery client moved on to the next assigned seller on their list. After 48 h with no response, the mystery client marked that seller as unresponsive. Some sellers responded after this period, in which case mystery clients proceeded with the interaction. Mystery clients did not have set “office hours,” texting with sellers during evenings, early mornings, and weekends. Some sellers requested video calls before engaging further; mystery clients refused these requests and in each situation, the seller nevertheless continued to engage with the mystery client. After beginning exchanges with our mystery clients, two sellers stopped responding. Mystery clients declined to pursue purchases from four sellers for various reasons: one made threatening and sexually explicit comments which made the mystery client feel unsafe, one asked for a prescription, one quoted prices that were far above the amount expected, and one said they would only sell in-person with cash payment.

Before contacting a seller, mystery clients reviewed the seller’s listing data and website or profile. Some websites gave instructions for what information clients should provide in their first message, such as approximate gestational age and location where they lived. In order for mystery clients to convincingly provide information requested by sellers, we developed profiles, or “scripts” for them to use. All mystery clients were to say that they did not know how far along in their pregnancy they were, only that their last menstrual period was six weeks before the date on which they contacted the seller and that they had not taken a pregnancy test; mystery clients were supposed to ask the sellers how pregnant they were. We had created profiles including marital status, age, and number of children; no sellers asked the mystery clients for their age or marital status. A small number asked whether the mystery client had ever given birth previously, but not the number of children they had.

Mystery clients filled in a self-administered structured questionnaire on their interaction with each seller, including information given, price paid, and drugs received. Once completed, these forms were uploaded and stored in SurveyCTO. Mystery clients also took screen shots of their text conversations with sellers, which were stored in a secure folder with the name of the seller’s ID in order to link data from the completed survey form to the logs of the interactions. To ensure all texts would be searchable, mystery clients also selected the text messages on a screen and pasted it into a Word document.

### Purchasing drugs offered by the seller

Most sellers offered “packets” of drugs, rather than misoprostol alone. When sellers offered a choice between various packets, we instructed mystery clients to ask for the seller’s recommendation. Mystery clients were instructed to ask what the other pills were for and what they do; they asked the seller to advise them on how many misoprostol pills to get. If any of the indicated packets exceeded the allotted budget of 1,500,000 Indonesian rupiah (about 106 USD) per purchase, the mystery client bought the packet closest to the recommended option that fit the budget (see Table 3). Most of the packets cost between IDR 750,000–1,249,999 (~ USD 52–86) (66%), with a quarter costing less than IDR 750,000 (< USD 52), and 8% costing between IDR 1,250,000 and 1,500,000 (~ USD 87–106). Mystery clients were encouraged to attempt to negotiate the price; they were instructed to ask for a lower price once. Some sellers offered to sell fewer pills for a lower price; in these cases, the mystery clients asked whether this would still cause an abortion and if the seller said yes, then agree on that packet. Mystery clients only ordered using standard shipping, and, once they made the purchase, asked sellers to send the tracking number for the shipment.

**Table 3:** Prices for misoprostol packets bought during online mystery client study, 2019

	Number of misoprostol purchases	Percent of misoprostol purchases
Less than IDR 750,000 (USD 52*)	20	26.3
IDR 750,000–1,249,999 (USD 52–86)	50	65.8
IDR 1,250,000–1,500,000 (USD 87–106)†	6	7.9
**Total**	**76**	**100**

These costs reflect all charges paid to online misoprostol sellers including shipping, which 16 sellers itemized, separate from the price of the drugs. Shipping prices ranged from IDR 9000 to 45,000, or roughly USD 0.62–3.11. Packages that mystery clients paid for, but did not receive, are included in this table

*These currency conversion costs are based on the exchange rate in September 2019

^†^The maximum allowable price we determined we would pay was IRD 1,500,000 per sale

Mystery clients reached out to the seller again as soon as the pills arrived, typically texting them something like “What do I do now?” Of the 76 sellers from whom we purchased misoprostol, 12 stopped responding to mystery clients as soon as payment was sent, with some blocking the mystery client’s number. In all, we received drugs from 64 sellers in our sample. We describe the drugs received and information given by sellers in a separate paper [7].

## Discussion

Presenting this methodology facilitates others’ engagement with studying online access to misoprostol or, potentially, other controlled medications which are purchased online. Specifying the decisions our study team made will lower barriers for others who intend to design online studies of misoprostol/controlled medications. In so doing, our intention is to make it easier for others interested in this field to create rigorous protocols that anticipate the surprises we encountered and thereby reach defensible decision-points to allow for capturing the most likely conditions of misoprostol/controlled medication access online.

Where high internet usage and restrictive abortion laws coexist, a robust online market will likely result. We see clear evidence to support this claim in the hundreds of listings that turned up in our searches and the streamlined process sellers used with clients who reached out to them. On the other hand, the clandestine nature of the service provision coupled with the fluidity of the online market creates new challenges for women seeking abortion. As evidence from the study based on this protocol suggests, challenges for women seeking misoprostol from online sources include not receiving drug already paid for, receiving incorrect drugs, and difficulty obtaining information on the drugs provided [7]. Of the 76 purchases made, 64 drug packages were received, 73% contained misoprostol, and 47% contained at least 800 mcg of misoprostol [7].

Future work in this area would benefit from testing the medications that the drug sellers sent to increase certainty about the active ingredients in unmarked/expired pills. Interviewing online drug sellers would provide new insights into how the seller acquired the controlled medication, how they screen potential buyers, their knowledge regarding appropriate dosages and physical effects as well as side effects and potential complications that should be communicated to the purchaser, their knowledge about contraindications, their relationship with other online sellers (if any), whether it is their primary source of income, as well as how they manage their online presence (e.g. how many places to advertise, how often they have to take down a profile and change their URL, whether they have been targeted by law enforcement). In addition, longitudinally studying the flux in the availability of online misoprostol through monitoring the online environment would also enrich our understanding of women’s access to misoporstol through online sellers.

## Limitations of the method

This protocol identified as complete a list as possible of sellers in Indonesia offering to sell misoprostol online, linked individual sellers to their multiple listings, and systematically selected a sample of them to be contacted by mystery clients. Nevertheless, our study is subject to several limitations. First, we cannot be certain that the sellers we identified, even in a very exhaustive search of the platforms we chose, constitute the full universe of online misoprostol sellers in Indonesia. It is likely that our choice of platforms limited our search results. We did not include Facebook posts because it was prohibitively difficult to create a fake Facebook account. Other popular social media or community platforms that would not turn up in a web search, such as Discord, TikTok, Twitch, or Snapchat, may also be used, perhaps by different sellers who do not advertise on the platforms we searched.

In addition, we quickly discovered that even after carefully removing listings linked to the same seller, we still encountered potential overlap between sellers we had originally thought to be unique in our sample. This indicates that the supply side of this market may be more concentrated than we had initially perceived and raises the question about the degree of connection between sellers of misoprostol online. Part of the answer to this question may be found in how these sellers are procuring the drug, a topic we could not explore in this study but which should be explored in future studies.

Finally, due to constraints posed by the measure of size we used to select sellers, programmatic probability proportional to size sampling was not possible. Our objective for sampling was simply to obtain a random sample that made sure to include sellers with the largest online presence, in a reproducible way. We were not trying to represent a population or detect differences between groups. Researchers undertaking similar studies which require any degree of statistical power should bear this in mind.

## Conclusion

As women seek to terminate unwanted pregnancies in legally restrictive settings, online sales of misoprostol must be considered. With the Covid pandemic constraining movement, the importance of this way of procuring misoprostol will likely become more appealing. Understanding this unregulated landscape is important if we are to try to improve women’s ability to safely conduct an abortion in highly restrictive settings.

## Data Availability

The datasets generated and analysed during the current study are not publicly available due because they were not the primary endpoint of the study rather the data that informed the primary endpoints. The data are available from the corresponding author on reasonable request.
